# Human Deciduous Teeth Stem Cells (SHED) Display Neural Crest Signature Characters

**DOI:** 10.1371/journal.pone.0170321

**Published:** 2017-01-26

**Authors:** Karlen G. Gazarian, Luis R. Ramírez-García

**Affiliations:** Department of Medicine Genomics and Environmental Toxicity, Institute of Biomedical Research, Mexican National Autonomous University, Mexico City, University Campus, Mexico; Second University of Naples, ITALY

## Abstract

Human dental tissues are sources of neural crest origin multipotent stem cells whose regenerative potential is a focus of extensive studies. Rational programming of clinical applications requires a more detailed knowledge of the characters inherited from neural crest. Investigation of neural crest cells generated from human pluripotent stem cells provided opportunity for their comparison with the postnatal dental cells. The purpose of this study was to investigate the role of the culture conditions in the expression by dental cells of neural crest characters. The results of the study demonstrate that specific neural crest cells requirements, serum-free, active WNT signaling and inactive SMAD 2/3, are needed for the activity of the neural crest characters in dental cells. Specifically, the decreasing concentration of fetal bovine serum (FBS) from regularly used for dental cells 10% to 2% and below, or using serum-free medium, led to emergence of a subset of epithelial-like cells expressing the two key neural crest markers, p75 and HNK-1. Further, the serum-free medium supplemented with neural crest signaling requirements (WNT inducer BIO and TGF-β inhibitor REPSOX), induced epithelial-like phenotype, upregulated the p75, Sox10 and E-Cadherin and downregulated the mesenchymal genes (SNAIL1, ZEB1, TWIST). An expansion medium containing 2% FBS allowed to obtain an epithelial/mesenchymal SHED population showing high proliferation, clonogenic, multi-lineage differentiation capacities. Future experiments will be required to determine the effects of these features on regenerative potential of this novel SHED population.

## Introduction

Stem cells from human exfoliated deciduous teeth (SHED) [1] [2] [3], derived from adult wisdom teeth pulp [4], and periodontal ligament [5] attract wide attention owing to their multipotent stemness and regenerative potentials. Studies on animal embryos documented that dorsal neuroepithelial cells, orchestrated by a gene network [6] delaminate from the border between neural and non-neural ectoderm [7] via a partial epithelial-to-mesenchymal transition (EMT) and migrate as cranial wave migratory cells [8,9] to a plethora of developing tissues [10] [11] [12] [13]]. This ecto-mesenchymal, clonogenic, and multipotent [12] neural crest population was identified by verifying the expression of Sox10, p75, HNK-1, and Ap-2 genes [14] [15] [16] and by the expression of the genes required for their migration, ZEB, SNAIL1, SLUG, FOXD3 and others. Their path could be traced during embryogenesis by specific markers of postnatal sites, mouse pulp [17] and ligament [18]. In human embryogenesis, the same markers–p75, HNK-1, Ap-2–were detectable to the stages earlier than S20 [19] [20] but could not evidently provide evidence on movements of neural crest cells to their destinations. Hence, investigation of the neural crest markers in postnatal dental cells was the approach that was used. Several groups reported on expression of the neural crest marker p75 by a subset of dental cell populations, SHED [21] [22], third molars [23] [24], dental follicle [25] and periodontal ligament [26], as the evidence of their origin from neural crest. Recent studies on *in vitro* generation of neural crest cells from human pluripotent stem cells, embryonic (ESC) and induced (iPSCs) [27–33], described the specific culture conditions required for the phenotypic and gene expression characters of neural crest cells can be displayed. The studies showed that neural crest culture condition is distinct from that of dental cells. Optimal for neural crest cells is serum-free medium with activated Wnt and inhibited SMADs pathways, whereas optimal for expansion of dental cells is serum-rich (contain regularly 10% foetal bovine serum, FBS) medium without these specific signaling requirements. Respectively, when epithelial-like neural crest cells were transferred from their medium to the dental cell medium, they underwent an epithelial-to-mesenchymal transition losing their attributes [28] [29]. This gives rise to a possibility that epithelial-to-mesenchymal transition is inhibitory for the neural crest identity genes and that such a transition is induced both in neural crest and in dental cells by Tgf-β present in FBS [34] [35] [36]. We describe experiments demonstrating that under the culture conditions adequate for expression of neural crest characters a proportion of SHED undergo mesenchymal-to-epithelial transition and the cells become similar to neural crest cells.

## Materials and Methods

### Sample collection

Primary teeth of 8 children (7–8 years-old, three males and 5 females) were collected in odontological clinics of Mexico City, with written informed consent letters signed by their parents. Teeth were immediately placed in sterile Hank's Buffered Salt Solution (HBSS, Gibco) containing 2× Antibiotic-Antimycotic Solution (Anti-Anti, Gibco), and were transferred to laboratory and processed as soon as possible within 24 h. The use of the teeth in this study was approved by the Bioethics Committee of the Biomedical Research Institute (National Autonomous University of Mexico).

### Isolation and culture of SHED

The teeth were repeatedly washed with commercial mouthwash solution (Advanced Listerine) and then with 2× Anti-Anti in Phosphate Balanced Saline Solution (PBS, Thermo Fisher Scientific). The teeth were mechanically broken with a pincer to expose the soft pulp tissue, which was minced in sterile glass Petri dish and digested with 3 mg/mL Collagenase IV (Sigma-Aldrich) solution in PBS for 60 min at 37°C in a CO_2_ cell culture incubator. The enzyme was inactivated by dilution with DMEM/F12 (Thermo Fisher Scientific), the digested tissues were seeded in a 35-mm plastic dish (passage 0) and cultured either in the regular mesenchymal medium composed of DMEM/F12 supplemented with 10% FBS, or a medium defined as DentEpiMesMed containing DMEM/F12 basal medium containing 1xN2 supplement (Invitrogen), 100 μg/mL L-Ascorbic acid-2-phosphate, 50 μM β-mercaptoethanol, 1x Glutamax (Thermo Fisher Scientific), MEM Amino Acids Solution 1x (Invitrogen), 2.5 ng/mL bFGF (basic Fibroblast Growth Factor, Thermo Fisher Scientific), 10 ng/mL IGF-1 (Insulin Growth Factor, Thermo Fisher Scientific), 10 ng/mL EGF (Epidermal Growth Factor, Thermo Fisher Scientific), 1x Anti-Anti (Thermo Fisher Scientific). For different purposes, the medium was either serum-free or supplemented with 1% or 2% FBS. The serum-free (0% FBS) medium contained 2.5 ng/mL TGF-β2 (Peprotech). For the culture in the 1% FBS and in 0% FBS, the cells at the stage of isolation after the treatment with the enzyme were first cultured 2 days as suspensions and then transferred to fibronectin-coated plate. For the neural crest culture condition, the cells were isolated and incubated in DentEpiMesMed containing 2%FBS for two passages; these cells were transferred to serum-free condition not containing TGF-β2 and supplemented with 1μM BIO (Sigma-Aldrich) and 5 μM REPSOX (Sigma-Aldrich) for five days, then the cells in this medium were seeded in fibronectin-coated plate. At approximately 80% confluence, the cells were collected by 5 min digestion with TrypLE Express (Thermo Fisher Scientific) and seeded in p60 dish (passage 1).

### Morphological characterization of the cells

Morphological characterization of SHED was performed using an Olympus IX71 phase contrast microscope. Image analysis was performed using QCapture Suite software.

### Cell population doubling time

Passage 1 cells were trypsinized and 2 × 10^4^ cells were seeded per well of a 12-well plate, cultured in the DentEpiMesMed with 2% FBS medium, and counted at 24, 48 and 72 h of incubation. Three replicates of this procedure were performed; then, the cells from the last count were re-seeded in a 12-well plate, cultured in the medium and counted again after 24, 48, and 72 h of incubation. This procedure was done with the cells until five passages. The population doubling time (td) was obtained from the equation:

$$\ln A=\frac{\ln2}{td}t+\ln A_{0}$$ (1)

where *t* is the time of incubation, *A*_*o*_ is the number of cells at the initiation of the experiment (2 X 10^4^), and *A* is the number of cells at time *t*. The value for *td* was obtained from the slope of the linear regression of the curve ln*A* vs *t*(1). Standard deviations and linear regressions were obtained. GraphPad Prism software was used.

### Clonogenic assay

Approximately one hundred cells from passage 2 were seeded in a 100-mm dish and cultured for 10 days in DentEpiMesMed (2% FBS). Colonies were fixed with 4% paraformaldehyde (PFA, Sigma-Aldrich) and stained with 0.5% crystal violet. Colonies containing approximately 50 cells were included in the results. Two replicates of the experiment were performed.

### Immunocytochemistry

SHED and cells of an ESC line (ESI-BIO, USA) were seeded on glass chamber-slides and cultured until they reached 80% confluence. Cells were fixed with 4% PFA in PBS for 30 min at room temperature (RT) and permeabilized with 0.1% TritonX-100 in PBS for 10 min at RT. Non-specific binding was blocked with 1% bovine serum albumin (BSA, MP Biomedicals) in PBS for 2h at RT. The primary antibodies used were: anti-SOX2 (Genetex), anti-MASH-1 (Abcam), anti-Sox10 (Abcam), anti-Oct ¾ (Cell Signaling), anti-Nanog (Cell Signaling), anti TRA-1-61 (Stemgent), and anti-TRA1-81 (Stemgent). Antibodies were diluted in blocking solution and added to the cells, which were preliminarily washed three times with PBS, and incubated overnight at 4°C under dark conditions and then with Alexa488 or Alexa568-conjugated secondary antibodies (Invitrogen) at RT for 1 h. The anti-β-Tubulin conjugated with Alexa Fluor® 555 Mouse, ClassIII TUJ1 was obtained from BD Biosciences. Nuclear counterstaining was performed with Hoechst 33342 (Thermo Scientific) as recommended by the manufacturer. After extensive washing with PBS, coverslips were mounted onto slides. Stained cells were analyzed using an Olympus IX71 phase contrast fluorescent microscope and a confocal laser scanning microscope (Zeiss LSM5 Pascal). The immunocytochemical images were analyzed with an Olympus IX71 phase contrast fluorescent microscope and a QCapture Suite Software.

### End-point RT-PCR assay

Total RNA was isolated using Trizol (Thermo Fisher Scientific) followed by treatment with DNAase (Thermo Fisher Scientific) and a column clean-up step using the RNeasy Mini kit (Qiagen). Gene expression was analyzed by RT-PCR amplification using One-Step RT-PCR kit (Qiagen) and primers (Table 1). Beta-actin or GADPH were included as housekeeping genes and (RT-) as negative control for the reaction.

**Table 1:** Primers used in RT-PCR/RT-qPCR.

Gene	Gene accession number	Forward (5´-3´)	Reverse (5´-3´)	Product length (bp)
OCT4	NM_001285987.1	GACAGGGGGAGGGGAGGAGCTAGG	CCTCCAACCAGTTGCCCCAAACTCCC	140
SOX2	NM_003106.3	TGGCTCCATGGGTTCGGTGGT	GAGGGGCAGTGTGCCGTTAATG	232
NANOG	NM_024865.3	AAATTGGTGATGAAGATGTATTCG	GCAAAACAGAGCCAAAAACG	132
MYC	NM_002467.4	TGGTCTTCCCCTACCCTCTCAAC	GATCCAGACTCTGACCTTTTGCC	266
KLF4	NM_004235.4	CAAGTCCCGCCGCTCCATTACCAA	CCACAGCCGTCCCAGTCACAGTGG	227
HTERT	NM_198253.2	CCTGCTCAAGCTGACTCGACACCGTG	GGAAAAGCTGGCCCTGGGGTGGAGC	447
FABP4	NM_001442.2	GGCATGGCCAAACCTAACAT	TTCCATCCCATTTCTGCACAT	199
PPARG	NM_138712.3	CCACTTTGATTGCACTTTGGTACTCTTG	CTTCACTACTGTTGACTTCTCCAGCATTTC	134
ALP	X55958.1	GGACCATTCCCACGTCTTCAC	CCTTGTAGCCAGGCCCATTG	137
BGLAP	NM_001199661.1	CCCAGGCGCTACCTGTATCAA	GGTCAGCCAACTCGTCACAGTC	112
ACTB	NM_001101.3	TGGCACCCAGCACAATGAA	CTAAGTCATAGTCCGCCTAGAAGCA	186
CD44	NM_000610.3	CTGCCGCTTTGCAGGTGTA	CATTGTGGGCAAGGTGCTATT	109
SOX10	NM_006941.3	CCTCACAGATCGCCTACACC	CATATAGGAGAAGGCCGAGTAGA	161
SOX9	NM_000346.3	AGCGAACGCACATCAAGAC	CTGTAGGCGATCTGTTGGGG	85
SNAI1	NM_005985.3	CGGCCTAGCGAGTGGTTCTTCT	AGGCTTCCGATTGGGGTCGGAG	117
SNAI2	NM_003068.4	AGGAATATGTGAGCCTGGGCGC	TGCTCTGTTGCAGTGAGGGCAA	167
ZEB1	NM_001174096.1	CCCGCGGCGCAATAACGTTACAA	TTCCTGTGTCATCCTCCCAGCAGTT	224

### RT-qPCR assay

Quantitative reverse transcription–polymerase chain reaction (RT-qPCR) was performed using KAPA SYBR FAST (Kappa BioSystems) master mix on a Rotor-Gene6000 (Qiagen) thermal cycler. The relative expression was determined using Pfaffl’s relative quantitation method.

### Flow cytometry

For marker analysis, the cells were detached using Accutase (Thermo Fisher Scientific) and washed by adding PBS supplemented with 2 mM EDTA and 0.5% BSA. Cells were centrifuged at 300 g and 4°C, incubated for 15 min in Cytofix fixation buffer (Becton Dickinson), washed with PBS, and centrifuged under aforementioned condition. The pellets were re-suspended in an adequate volume of staining buffer comprised of PBS supplemented with 0.5% BSA and 2 mM EDTA. Cell concentration, antibody dilution, and incubation conditions were used as recommended in the antibody datasheet. The fluorochrome-conjugated antibodies were Anti-CD90-PE, Anti-CD105-APC, Anti-CD73-PE, Anti-CD271/p75-APC, Anti-HNK-1-FITC, Anti-E-CADHERIN/CDH1-APC, Anti-GFAP-A647, Anti-PSA-NCAM-APC, Anti-MAP2-A647, Anti-NESTIN-PE, Anti-IgG1-FITC, Anti-IgG1-PE, Anti-IgG1-APC (all from Miltenyi Biotec), DAPI (BD Biosciences), and Anti-Ki67-PE (BD Biosciences). Stained cells were washed by centrifugation at 300×g and re-suspended in 500 μL PBS supplemented with 2 mM EDTA. Sample acquisitions and analysis were done with an Attune™ flow cytometer (Thermo Fisher Scientific) and FlowJo software, respectively.

### Magnetic activated cell sorting (MACS)

Cells were sorted with MACS using human anti-p75 MicroBead kit (APC) as recommended (Miltenyi Biotec).

### Osteogenic differentiation

For osteogenic differentiation cells were seeded at a density of 5 × 10^3^ cells/cm^2^ in 6-well plates and cultured for 3 weeks in osteogenic differentiation medium comprised of DMEM-F12 basal medium supplemented with 10% FBS, 1× Anti-Anti, 0.1 μM dexamethasone, 50 μg/mL ascorbic acid (Sigma-Aldrich), 50 μg/mL ascorbate-2-phosphate (Sigma-Aldrich), 10 μM β-glycerol phosphate (Sigma-Aldrich), and 100 nM Vitamin D (Sigma-Aldrich). Calcium deposits were revealed by staining with Alizarin red S (Sigma-Aldrich) on day 21.

### Adipogenic differentiation

For adipogenic differentiation, cells were seeded at a density of 1 × 10^4^ cells/cm^2^ in 6-well plates and cultured for 3 weeks in adipogenic differentiation medium comprised of DMEM/F12 supplemented with 10% FBS, 1× Anti-Anti, 1 μM dexamethasone (Sigma-Aldrich), 200 mM indomethacin (Sigma-Aldrich), 10 μM insulin (Sigma-Aldrich), and 0.5 mM 3-isobutyl-1-methylxanthine (Sigma-Aldrich). Culture medium was replaced twice a week. Adipogenic differentiation was determined by Oil red (Sigma-Aldrich) staining on day 21.

### Neurogenic differentiation

The cells at a density of 1 × 10^5^ cells/cm^2^ were seeded on Matrigel (BD Biosciences) coated plates with Sox2-inducing medium [31] containing the Optimem 20 ng/mL bFG2, 20 ng/mL EGF, 2 μM SB43152, 1 μM LDN-193189 (Sigma), 1× N2, 1× B27, and 10 ng/mL Leukemia Inhibitory Factor (LIF; Peprotech). On the third day, the medium was switched to neural induction medium [29] containing the Neurobasal-A medium (Thermo Fisher Scientific) supplemented with 1× N2 supplement (Thermo Fisher Scientific), 20 ng/mL bFG2, 10 ng/mL Brain Derived Neurotrophic Factor (BDNF, Peprotech), 10 ng/mL Glial-Derived Neurotrophic Factor (GDNF, Peprotech), 10 ng/mL Nerve growth factor (NGF, Peprotech), 10 ng/mL Neurotrophin-3 (NT-3, Gibco), 200 μM ascorbic acid (Sigma-Aldrich), 10 μM forskolin (Sigma-Aldrich), 250 μM 3-Isobutyl-1-methylxanthine (IBMX, Sigma-Aldrich) 2 μM SB43152(Sigma-Aldrich), and 1 μM LDN-193189(Sigma-Aldrich).

## Results

### Epithelial-like cells expressing p75 and HNK-1 emerge in SHED population cultured in serum-free and in 1%FBS-containing medium

**Fig 1:**
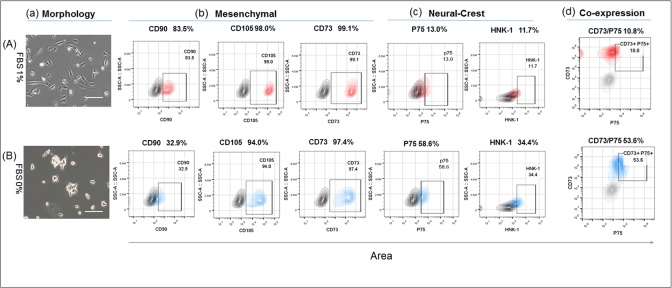
Morphological and gene expression characteristics of SHED cultured in 1%FBS and 0%FBS DentEpiMesMed. (a)—bright field images of the cells in 1%FBS medium and in 0%FBS medium (2.5ng/ml of the TGFβ was added), respectively. Scale bars 50μm; (b)(c)—number of the cells expressing mesenchymal (CD90, CD105, CD73) and neural crest (p75, HNK-1) genes in medium containing 1% FBS and 0% FBS, respectively; (d)–number of the cells co-expressing CD73 and p75 genes in 1%FBS and 0% FBS medium. Hear and below see Material and Methods section for composition of DentEpiMesMed.

SHED cultured in the regular serum-rich (10% FBS) expansion medium were, as previously described [1] [2] [3], typical [37] fibroblast-like mesenchymal population (S1A Fig), in which over 94% of the cells expressed CD73, CD105, and CD90 (S1B Fig), and only around 2% were positive for the classical [31] neural crest markers p75 and HNK-1 (S1C Fig and Table 2) confirming the earlier description of p75 in 4% to 10% of dental pulp [21] [22] [23] [24], follicle [25] populations, and in, approximately, 1% of periodontal ligament cells [26]. Human pluripotent stem cell-derivative neural crest cells have been shown to lose their epithelial morphology and the markers at a similar serum-rich condition [28]. To test the possibility that this culture condition inhibited neural crest attributes in dental pulp mesenchymal cells, we cultured them in serum-deficient and serum-free medium. We designed a modified medium called DentEpiMesMed (see Material and Methods) and supplemented it with FBS at various concentrations below 10% or used serum-free (0% FBS). EGF and bFGF were included to compensate the eliminated serum growth factors [38] and BIO + REPSOX were added in some of the experiments as neural crest requirements. Using these media, we drew the cells gradually near the serum-free neural crest culture condition [28] [29]. At FBS concentrations equal to or below 2%, mesenchymal SHED cultures contained variable numbers of epithelial-like cells, and the marker analysis by flow cytometry assay revealed the expression of p75 and HNK-1 at a higher proportion of cells (Fig 1C and Table 2). Thus, in serum-free medium, SHED were phenotypically epithelial-mesenchymal populations containing up to 58% of p75 and 34% of HNK-1. The same analysis showed that the cells that acquired an epithelial morphology co-expressed p75 with mesenchymal markers, CD73 and CD105 (since about 94% of the cells were positive for these markers). Additionally, a two-color assay showed over 50% of the cells co-expressing p75 and CD73 (Fig 1Bd). This was a remarkable finding in light of the co-expression of p75 and CD105 by neural crest cells [28]. Unlike insensitivity of CD73 and CD105 to low serum/serum-free condition, CD90 gene was drastically downregulated, showing in this respect a similarity to the neural crest genes. In concrete terms, the number of the cells expressing CD90 was decreased several-fold (Fig 1B), demonstrating a strong dependence on serum concentration, as did the neural crest p75 and HNK- genes. Table 2 presents overall data on the reproducibility and the range of variations from eight experimental settings.

**Table 2:** Flow cytometry analysis of mesenchymal and neural crest markers in SHED cultured at different concentration of FBS (n = 8).

%FBS	Cell Surface Markers			
	Mesenchymal		Epithelial-Like (Neural Crest)	
	CD73	98.9(±1.0)	P75	2.5(±1.5)
10	CD90	97.5(±2.0)	HNK-1	1.0(±0.5)
	CD105	97.9(±1.5)		
	CD73	95(±2.0)	P75	15.7(±9.5)
2	CD90	92(±5.0)	HNK-1	5.2(±3.5)
	CD105	96(±4.0)		
	CD73	95(±3.5)	P75	43.4(±18.5)
0	CD90	32(±20.5)	HNK-1	22.7(±13.5)
	CD105	96(±3.5)		

### Culture of purified p75+ and p75^-^ SHED fractions confirmed the serum-dependent phenotypic and marker modulations

**Fig 2:**
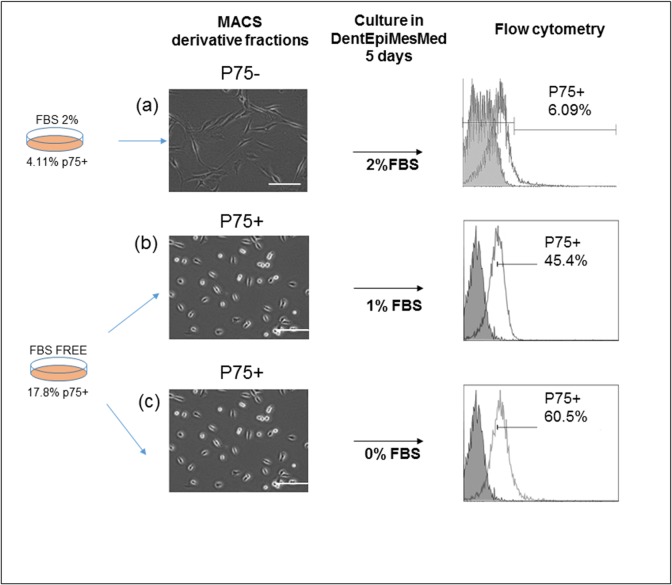
Phenotypic and p75 instability in the cells at serum-free and at low FBS concentrations. (a)—SHED containing 4.1% p75^+^ cells in DentEpiMesMed (2%FBS) were fractionated by MACS using an anti-p75 antibody into p75-positive and p75-negative fractions; the p75-negative cells were cultured five days in the same medium and the amount of the cells with the marker were determined by flow cytometry. (b), (c)—SHED containing 17.8% p75^+^ cells in the 0%FBS DentEpiMesMed (2.5ng/ml of the TGFβ was added) were used to sort out the p75^+^ cells which were cultured five days under two conditions: 1%FBS (b) and 0%FBS (2.5ng/ml of the TGFβ was added) (c); the proportion of the p75^+^ cells were determined by flow cytometry.

Previously, it has been shown that neural crest cells can be purified from ESC- or iPSC-derived neuroepithelial cultures using antibody to p75 [28]. Similarly, this specific antibody was used to isolate a low number of p75+ cells from different dental populations cultured in regular 10% FBS medium [22] [23]. Using this specificity antibody, we separated the p75-positive cells from the p75-negative cells of two SHED populations cultured in (i) low-serum (2% FBS) medium and (ii) FBS-free medium, which, according to flow cytometry analysis, contained 4% and 17.8% of p75+ cells, respectively (Fig 2, left). Morphologically, the p75-positive cells were epithelial-like and p75-negative ones were fibroblast-like cells. Culturing for 5 days (a single passage period) of the p75-negative fraction in 2% FBS medium resulted in 6% newly emerged p75+ cells and the p75+ fractions cultured in 1% FBS or in FBS-free medium led to significant (45.4% and 60.5%, respectively) loss of the marker during same five days. Noteworthy, Mikami et al. [22] purified a subset of 2% p75+ component of SHED grown in 10% FBS medium, cultured in the same medium and observed the loss of the p75 marker. Confirming this observation on the loss of this marker, we additionally observed an opposite event–the emergence of the p75 marker in 6% of the p75-negative fraction, highlighting an extreme epigenetic instability (activation/inactivation) of p75 gene in the serum-deficient/serum-free condition. Additionally, the result of these experiments showed that expression of genes regulating the morphological traits was unstable, leading the cells to epithelial-like or fibroblast-like phenotypes. Both the marker and phenotype changes indicated that this FBS condition favored more the epithelial-to-mesenchymal transition and inactive p75 gene.

### Serum-free medium containing neural crest culture requirements reinforced the emergence of epithelial-like cells, upregulated the p75, Sox10, E-cadherin genes and downregulated EMT genes

**Fig 3:**
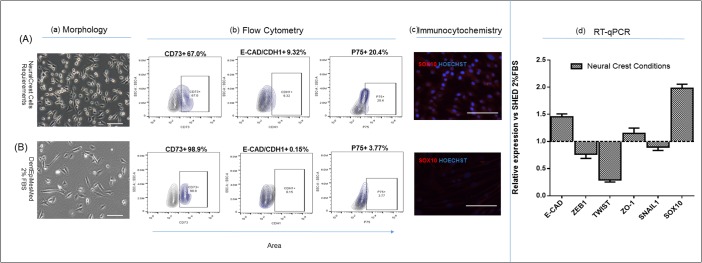
Characteristics of SHED cultured in neural crest vs. 2%FBS conditions. The cells were isolated and cultured for two passages in 2% FBS-containing DentEpiMesMed (B) and a sample of the cells were cultured as suspension for five days in serum-free medium containing 1μM BIO and 5 μM REPSOX then seeded in fibronectin-coated plate (A). The properties of the cells grown in the two conditions were determined. (a)–bright field images comparing the morphology of the cells on (A) and (B); (b)–flow cytometry evaluation of the SHED populations expressing mesenchymal (CD73), epithelialization (E-cadherin) and neural-crest (p75) genes. (c) immunofluoresence assay of Sox10 in (A) and (B); (d) RT-qPCR-determined relative transcription levels of E-cadherin, ZEB1, TWIST, ZO-1, SNAIL1 and SOX10 genes.

Menendez and coworkers [28] were the first to show that serum-free medium containing inducers of Wnt signaling in an inactive SMAD 2/3 context was the optimal condition for the neural crest identity characters. To meet these requirements, BIO (upregulates Wnt signaling through inhibition of GSK3b) and REPSOX (a TGF-βR1 inhibitor) were used in this study. We compared effects on SHED characters of the culture in two DentEpiMesMed (see Material and Methods) conditions: (i) with 2% FBS and (ii) serum-free supplemented with BIO and REPSOX, and observed contrasting effects (Fig 3). The morphology of the cells was fibroblast-like in the presence of FBS and epithelial-like in the neural crest medium (Fig 3A). The neural crest medium, as showed flow cytometry assay, decreased significantly the frequency of the mesenchymal marker CD73, upregulating E-cadherin responsible for the epithelialization, and p75; immunofluorescence assay showed upregulation of SOX10. Concurrently, the qRT-PCR analysis indicated that the neural crest culture condition upregulated E-cadherin, ZO-1, SOX1O genes and downregulated EMT genes (ZEB1, TWIST, SNAIL 1), thus supporting our suggestion that MET/EMT Program induced by the used culture conditions modulated the functional state of neural crest-specific genes in SHED. These results presented unequivocal evidence that a large number of SHED were capable to respond to neural crest culture condition by activating their signature traits.

### SHED stemness and differentiation characteristics are maintained in 2% FBS-DentEpiMesMed

**Fig 4:**
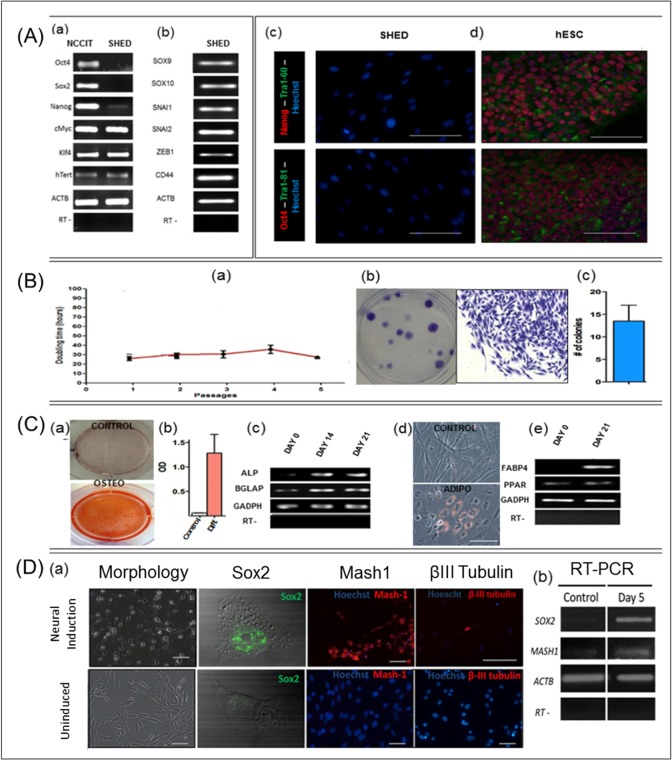
General characteristics of SHED population cultured in DentEpiMesMed 2%FBS. **A.** RT-PCR analysis of expression of pluripotency (a) and neural crest migratory (b) genes; RNA of NCCIT, an embryocarcinoma line (ATCC), was included as a pluripotency control in (a); ACTB gene and (RT-) served as reaction controls. Immunofluorescence assay of pluripotency markers of SHED (c), cells from the human ESC line (ESI-BIO, US) were included as positive control (d). **B.** Proliferation rate of SHED measured during five passages (a), and clonogenicity (b, c) of the cells. **C**. Osteogenic and adipogenic differentiation potentials of the cells. Osteogenic potential: (a)- calcium deposits detected by staining with Alizarin Red S; (b)- quantitative evaluation of the Alizarin Red S intensity by spectrophotometry (Epoch, Biotek)) in control and in differentiated (*diff*.) cultures; (c)- RT-PCR analysis of the transcription of ALP and BGLAP genes. GAPDH and RT- were positive and negative controls, respectively. Adipogenic potential: (d)—lipid droplets in adipocytes; (e)- RT-PCR assay of the expression of FABP4 and PPAR genes. **D.** Neuronal lineage potential. (a)–morphological changes, expression of SOX2, MASH-1 at day 5 and βIII-tubulin at day 14 determined by immunofluorescence analysis; (b)—RT-PCR analysis of transcription of SOX2 and MASH-1 genes. GAPDH and RT- were positive and negative controls, respectively.

The above results indicated that SHED population at low-serum condition (2% FBS) contained an epithelial-like fraction expressing p75 and HNK-1. Considering this SHED epithelial-mesenchymal population resembling neural crest, useful for basic and applied research, we investigated its stemness and the multipotent differentiation potentials. The data in (Fig 4Ab) shows that, in addition to p75 and HNK-1, cells expressed the specific migratory genes SOX9, SOX10, SNAI1, SNAI2, ZEB1, and CD44. The expression of these genes, especially those of the SOX family, supports the notion that these postnatal stem cells retained the markers of their neural crest migratory ancestors. Remarkably, the expression of the pluripotency genes (Oct3/4, NANOG, TRA-1-81, TRA-1-61) was undetectable by the used methods, which was not surprising in case of the multipotent cells (Fig 4Aac), although we cannot exclude the possibility that higher resolution techniques could detect the expression of these genes as was shown for dental follicle cells [25]. The addition of bFGF and EGF to compensate their deficit in this 2% FBS medium supported fairly high proliferation rate (doubling time 20–25 h, Fig 4Ba) clonogenic efficiency 14%, Fig 4Bbc). The cells expanded in this medium and transferred to specific induction media showed signs of osteogenic (Fig 4Ca,b,c), adipogenic (Fig 4Cd,e), and neuronal (Fig 4D) differentiation. We paid special attention to the neuronal lineage for its more relatedness to neural crest characters and observed activation prior to βIII-Tubulin, of SOX2 and MASH-1 genes (Fig 4Da) shown to be required for the commitment to peripheral neuronal differentiation [26]. To our knowledge, this is the first induction of these genes in dental cells and was, we suppose, due to the expression of p75 and HNK-1. The observed downregulation of pro-neural NESTIN and glial GFAP genes, and upregulation of neuronal PSA-NCAM and MAP2 genes (S2A Fig) can be associated with the preferred neuronal differentiation. Altogether, these characteristics qualify SHED grown under this low-serum condition to be of a great utility for preclinical and clinical studies.

## Discussion

Previous convincing evidence from tracing experiments demonstrated that embryonic migratory neural crest cells are progenitors of mouse dental pulp [17] and periodontal ligament [18] cells. Human neural crest cells cannot be traced to their postnatal destination, hence searching for the signs of the neural crest identity in postnatal dental cells is a possibility that was used. Thus, several groups [22] [23] [26] described p75-marker in 4% to 10% of dental populations. The neurotrophin receptor p75(NTR) is expressed by adult neural cells playing a fundamental role in the development and maintenance of the nervous system [39]. In neural crest cells, p75 is one of the key markers so that an antibody to p75 alone purifies neural crest cells from the culture of ESC-derived neuroepithelal cells [27] and from dental pulp populations ([22,23] and this study). *In vitro* experiments with neural crest cells derived from human pluripotent stem cells have revealed that their identity characters, including p75, are suppressed in mesenchymal medium containing 10% FBS [28]. We suggested that dental cells, SHED in particular, might be deprived of the neural crest traits in regular medium containing a similar high concentration of serum. The results of the study proved this suggestion showing that the set of the neural crest signature traits, that were observed previously, *in vivo*, in human embryos [20] and, *in vitro*, in human pluripotent stem cell-derivative neural crest cells [28], p75, HNK-1, SOX 10, can be activated in SHED. The role of these genes in dental cells remains to be elucidated. One possibility to verify in further experiments is that the activity of neural crest genes, specifically the p75 neurotrophin receptor, is required for inhibition of mesenchymal lineages [22] to ensure the neuronal lineage commitment via activation of SOX2-MASH-1 genes [30,31]. The other finding in this study which merits a deeper investigation is the heterogeneity of SHED regarding the responsiveness to the inducers of phenotypic and gene expression changes. While a small component of SHED population express p75 and HNK-1 genes even in the presence of 10% FBS ([22,23,26] and Table 2), a significant part of the SHED population retained mesenchymal phenotype in serum-free neural crest medium (Fig 3). At low FBS concentrations (1%, 2%), some of the cells underwent transition to the epithelial state and expressed p75 and HNK-1, but upregulation of SOX10 depended on the signaling requirements. The 2% FBS concentration was a cut-off point (threshold) for inter-transition of mesenchymal and epithelial neural crest markers. Repeated transferring of the cells to serum conditions above this threshold led to mesenchymal state and inactivity of the neural crest markers; conversely, serum concentration below this level led to epithelial state and upregulation of the neural crest genes. Both neural crest cells [30] and dental cells with epithelial phenotype co-expressed the neural crest (p75 and HNK-1) and mesenchymal (CD73 and CD105) genes, which may facilitate the MET and EMT transitions. Coexpression of p75 and mesenchymal markers was also observed in the cells from third molars by Alvares et al [23]. Since exogenously activated Wnt signaling, in the context of inactive TGFβ (SMAD 2/3), was required for upregulation of the key neural crest signature gene SOX10 [28], one can suppose that a low level of endogenous Wnt signaling in our SHED upregulated the p75 and HNK-1 genes in low-serum (below 2% FBS) and serum-free medium. The observed phenotypic and gene expression instability reflects the plasticity of the neural crest derivative lineages as demonstrated on Schwann cells [40], in melanocytes [41] and dental cells converting to melanocytes [42]. Studies on regenerative potential in preclinical experiments [43] [44] [45] [46] [47], banking of “clinical grade” dental stem cells [48] and trials [49] are in progress. Future identification of the factor(s) inducing EMT and MET will permit to employ them for modulating neural crest identity genes and test their role in regenerative applications of dental cells.

In conclusion, this study demonstrates that epithelial-like phenotype and the neural crest signature genes can be activated in a half of SHED providing further insights into the functional state of these genes in the dental cells.

## Supporting Information

S1 Fig Morphological and gene expression characteristics of SHED cultured in 10%FBS. (a) Bright field image showing mesenchymal morphology of a SHED population cultured in DentEpiMesMed supplemented with 10% FBS: determined by flow cytometry mesenchymal(b) (CD90, CD105, CD73) and neural crest markers(c) (p75, HNK-1), respectively.
(TIF)

S2 Fig Expression of neuronal lineage markers before and after neural induction. Cells grown in DentEpiMesMed (2%FBS)were induced to neural lineage in the Sox2-inducing medium(31); at the fifth day postinduction expression of NESTIN, GFAP, PSA-NCAM, MAP2 were determined by flow cytometry, (A) induced; (B) uninduced; the data in (A) coincide with the expression of SOX2 and MASH-1 genes(Fig 4D).
(TIF)
