# Supplementary figures and images for: Human Deciduous Teeth Stem Cells (SHED) Display Neural Crest Signature Characters
Source: PLoS One. 2017 Jan 26;12(1):e0170321. doi: 10.1371/journal.pone.0170321 (PMC5268458; doi:10.1371/journal.pone.0170321)

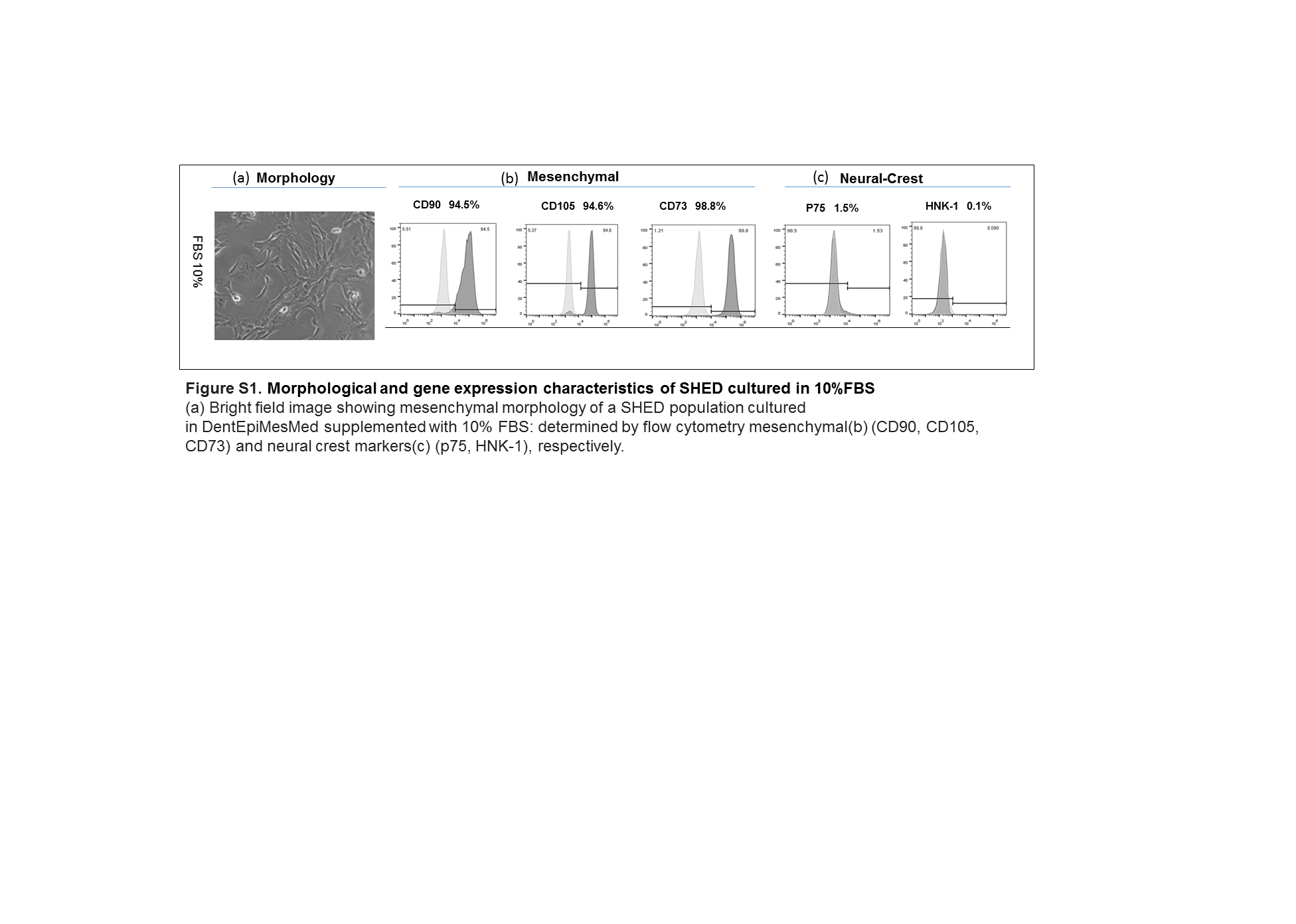

Supplement: S1 Fig — (a) Bright field image showing mesenchymal morphology of a SHED population cultured in DentEpiMesMed supplemented with 10% FBS: determined by flow cytometry mesenchymal(b) (CD90, CD105, CD73) and neural crest markers(c) (p75, HNK-1), respectively. (TIF) [file pone.0170321.s001.tif]

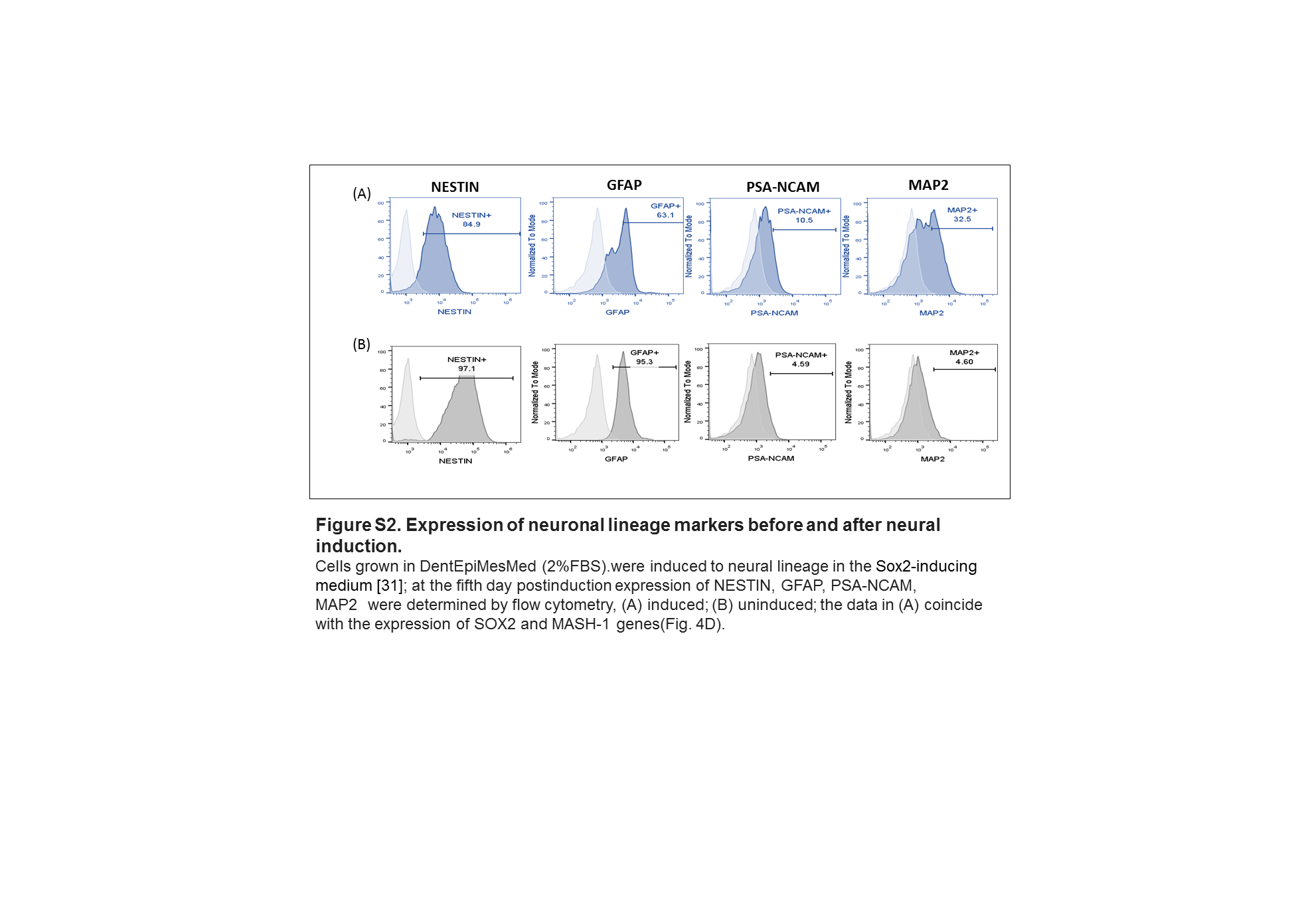

Supplement: S2 Fig — Cells grown in DentEpiMesMed (2%FBS)were induced to neural lineage in the Sox2-inducing medium(31); at the fifth day postinduction expression of NESTIN, GFAP, PSA-NCAM, MAP2 were determined by flow cytometry, (A) induced; (B) uninduced; the data in (A) coincide with the expression of SOX2 and MASH-1 genes(Fig 4D). (TIF) [file pone.0170321.s002.tif]
